# GPCRs in Autocrine and Paracrine Regulations

**DOI:** 10.3389/fendo.2019.00428

**Published:** 2019-07-12

**Authors:** Lap Hang Tse, Yung Hou Wong

**Affiliations:** ^1^Division of Life Science, Biotechnology Research Institute, Hong Kong University of Science and Technology, Hong Kong, Hong Kong; ^2^State Key Laboratory of Molecular Neuroscience, Molecular Neuroscience Center, Hong Kong University of Science and Technology, Hong Kong, Hong Kong

**Keywords:** G protein, receptor, autocrine, paracrine, signaling

## Abstract

G protein-coupled receptors (GPCRs) constitute the largest superfamily of integral membrane protein receptors. As signal detectors, the several 100 known GPCRs are responsible for sensing the plethora of endogenous ligands that are critical for the functioning of our endocrine system. Although GPCRs are typically considered as detectors for first messengers in classical signal transduction pathways, they seldom operate in isolation in complex biological systems. Intercellular communication between identical or different cell types is often mediated by autocrine or paracrine signals that are generated upon activation of specific GPCRs. In the context of energy homeostasis, the distinct complement of GPCRs in each cell type bridges the autocrine and paracrine communication within an organ, and the various downstream signaling mechanisms regulated by GPCRs can be integrated in a cell to produce an ultimate output. GPCRs thus act as gatekeepers that coordinate and fine-tune a response. By examining the role of GPCRs in activating and receiving autocrine and paracrine signals, one may have a better understanding of endocrine diseases that are associated with GPCR mutations, thereby providing new insights for treatment regimes.

## Introduction

To maintain homeostasis in humans, a wide array of extracellular factors is required to harmonize physiological activities between various organs and tissues. These signaling molecules in the form of hormones, peptides, neurotransmitters, proteins, ions, and lipids act via specific receptors to elicit cellular responses. Among the different receptor families, more than 700 G protein-coupled receptors (GPCRs) form the largest and the most diverse receptor superfamily that participate in virtually all aspects of human physiology. Most human GPCRs can be grouped into five families (Glutamate, Rhodopsin, Adhesion, Frizzled/Taste2, and Secretin) according to their structural similarity, which is known as the GRAFS classification system (1). The physiological relevance makes GPCRs one of the most popular drug targets (2), and their importance in the field of endocrinology is highlighted by the identification of naturally occurring GPCR mutations in patients with various endocrine diseases (3). In classical endocrine systems, hormones are released into the bloodstream and they modify target cells in a distant part of the body; it has become apparent that these processes are regulated by cellular communications encompassing autocrine, paracrine, intracrine, and juxtacrine interactions. In this review, we will focus on our current, yet evolving understanding of the autocrine and paracrine signals regulated by GPCRs in various physiological systems. Since signals mediated by GPCRs are regulated by a myriad of complex determinants, with crosstalk between different signaling pathways that culminate in the fine-tuning of cellular responses, it is important to examine the role of GPCRs in the context of signal processing within and between different cells and tissues.

## GPCRs and the Hierarchy of Endocrine, Autocrine and Paracrine Signaling

**Figure 1:**
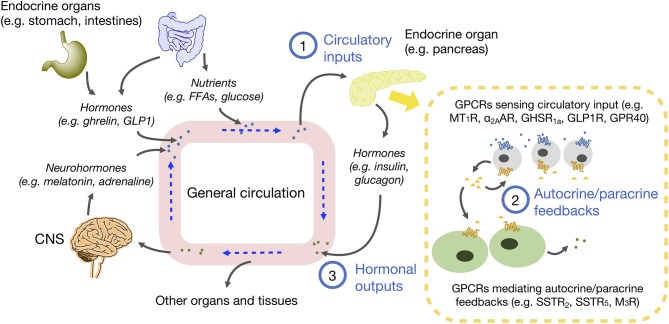
Schematic diagram of endocrine, autocrine, and paracrine communications in energy homeostasis. Circulating molecules such as neurohormones, hormones and nutrients are derived from CNS, endocrine organs and tissues, and small intestine. These molecules in blood are circulated to different parts of the body. The molecules diffused into endocrine organs act as circulatory inputs (1), which then bind to receptors on target cells and trigger the secretion of autocrine and paracrine factors, so as to foster communications between the same or different cell types (2). As a result of signal integration from various autocrine/paracrine factors, hormone secretions from endocrine cells are adjusted, and hormonal outputs are released into the circulation (3), which can further influence other organs and tissues to maintain homeostasis.

Although the discovery of autocrine and paracrine interactions was initially overshadowed by the characterization of endocrine glands, the concept of cells being able to secrete regulatory factors was first appreciated more than 200 years ago by leading scientists of the time, including Brown-Séquard whom many regarded as the “father of endocrinology.” It is now firmly established that the endocrine glands are regulated by a plethora of internal and external signals via blood circulation, and that these input signals can further trigger the release of autocrine/paracrine messengers. Various autocrine/paracrine factors are known to contribute to the communications and intricate feedbacks between different types of cells within an endocrine gland, resulting in a coordinated hormonal output and the corresponding physiological outcome. Remarkably, the same chemical molecules can be used in multiple contexts of endocrine, paracrine or autocrine signaling, or even in synaptic signaling. The function of these signaling molecules can be considered in a hierarchical manner (Figure 1) for the majority of endocrine organs as: (1) a circulatory input that initiates the subsequent autocrine/paracrine interactions; (2) an autocrine/paracrine messenger that mediates the feedback networks among different cells within the endocrine gland; and (3) a hormonal output secreted by endocrine cells, which enter the circulation and further serve as a circulatory input for other organs. Among these signaling processes, GPCRs are ineluctable mediators in sensing both circulatory inputs and autocrine/paracrine factors. According to the “GRAFS” classification scheme, there are five classes of vertebrate GPCRs (1) whose differential distribution on different cell types helps to ensure specificity of the feedback network. Because optimal utilization of nutrient is a vital prerequisite for survival, this review will focus on examples of GPCRs in the Rhodopsin and Secretin families that regulate energy homeostasis. However, it should be noted that the concept of GPCRs mediating autocrine/paracrine responses is applicable to many other endocrine tissues/organs such as adipose tissue (4), the adrenal gland (5), and testis (6). Other classes of GPCRs are also functionally essential for various endocrine systems; a prime example is the calcium-sensing receptor of the Glutamate family for calcium homeostasis (7).

## Pancreatic GPCRs in the Regulation of Energy Homeostasis

**Figure 2:**
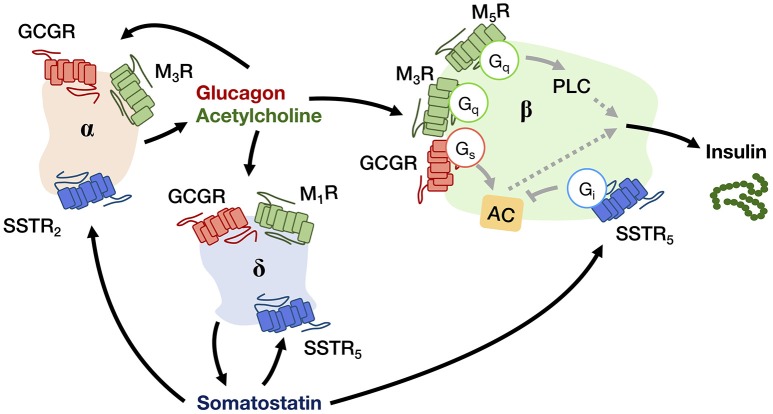
A schematic diagram showing the autocrine and paracrine interactions between pancreatic α-, β-, and δ-cells that regulate the insulin output. Glucagon and acetylcholine from α-cell (beige), and somatostatin from δ-cell (blue) are stimulatory and inhibitory signals for hormone secretion, respectively, that can act in both autocrine and paracrine manners via their receptors (GCGR for glucagon; M_1_R, M_3_R, and M_5_R for acetylcholine; SSTR_2_ and SSTR_5_ for somatostatin). The insulin output from β-cell (green) is adjusted by an integration of paracrine signals from both α- and δ-cells within the niche.

The human pancreas is composed of granular tissues embedded with a duct system. While exocrine cells constitute the major biomass of the pancreas, a small cluster of endocrine cells forms the pancreatic islet, including three key cell types: glucagon-secreting α-cells, insulin-secreting β-cells, somatostatin-secreting δ-cells (8) (Figure 2). The pancreatic islet is an important peripheral endocrine gland for maintaining blood glucose level and energy homeostasis. To adjust energy fluctuation caused by food intake, circadian rhythm or physical activities, the islet is sensitive to signals which are regulated by the hypothalamus as well as to other circulatory signals such as nutrients and hormones. Human islet GPCR mRNA profiling has identified 293 islet GPCRs that respond to 271 different endogenous ligands, of which at least 131 ligands are present in islet cells (9). However, the majority of islet GPCRs have unknown effects on pancreatic hormone secretion. Readers may refer to other reviews for the full list of islet GPCRs discovered in humans and their comparative analysis with mouse islet GPCRs (9, 10). Besides GPCRs, other receptor types such as tyrosine kinases are also able to regulate pancreatic responses. It should be noted that signal crosstalk and transactivation linkages between GPCR and non-PCR pathways constitute yet another layer of signaling complexity in energy homeostasis (11).

### GPCRs for Sensing Circulatory Inputs

There are numerous GPCRs in the pancreas that can detect circulatory nutrients and related hormones, and the major examples are listed in Table 1. A few examples will be discussed to illustrate the role of GPCRs as circulatory signal sensors that induce subsequent autocrine/paracrine interactions in the pancreas. One of the best-known incretin hormones is glucagon-like peptide 1 (GLP1), which is secreted by the intestinal L-cells upon food consumption and circulates throughout the body, including the pancreatic islet which expresses GLP1 receptor (GLP1R). GLP1R is coupled to G_s_ proteins to control the secretion of insulin, glucagon, and somatostatin that facilitate glucose disposal. The activation of GLP1R on mouse β-cells induces a robust up-regulation of insulin-like growth factor 1 (IGF1) receptor, which triggers the IGF1/IGF2 receptor autocrine loop associated with an increase of Akt phosphorylation, with the Akt pathway bestowing an anti-apoptotic effect (25). GLP1R expressed on β- and δ-cells can also direct the paracrine regulations by activating the secretion of insulin and somatostatin, respectively, to inhibit glucagon secretion by α-cells. Gastric inhibitory polypeptide (GIP), another incretin hormone which acts on the G_s_-coupled GIP receptor (GIPR), mediates similar responses as GLP1 but its mechanism of action is less understood (12). In addition, GIP can induce the production of interleukin-6 (IL6) by α cells, which in turn stimulates the production of GLP1 and insulin secretion by β-cells, forming another paracrine loop (24).

**Table 1:** Examples of GPCRs in the pancreatic islet for receiving circulatory inputs.

**GPCR**	**Family**	**Ligands**	**Transduction mechanism**	**Functions**	**References**
GHSR_1a_	Rhodopsin	Ghrelin	G_q/11_; G_i/o_; G_12/13_	• Inhibit insulin secretion^b^	(12)
GPR40	Rhodopsin	Long-chain carboxylic acid	G_q/11_; G_s_; G_i/o_	• Stimulate glucagon secretion^c^ • Enhance insulin secretion^a^	(13, 14)
GPR119	Rhodopsin	N-oleoylethanolamide	G_s_	• Enhance insulin secretion^c^ • Stimulate β-cell replication^c^	(15, 16)
ChemR23	Rhodopsin	Chemerin	G_i/o_	• Stimulate glucagon secretion^c^ • Enhance insulin secretion^a^	(17)
MT_1_	Rhodopsin	Melatonin	G_i/o_; G_q/11_	• Stimulate glucagon secretion^a^ • Enhance insulin secretion^b^	(18)
MT_2_	Rhodopsin	Melatonin	G_i/o_	• Stimulate glucagon secretion^a^ • Enhance insulin secretion^b^	(18)
α_2A_AR	Rhodopsin	Adrenaline; Noradrenaline	G_i/o_; G_s_	• Inhibit insulin secretion^b^	(19)
β_1_AR	Rhodopsin	Adrenaline; Noradrenaline	G_s_; G_i/o_; β-arrestin $\frac{1}{2}$	• Stimulate glucagon secretion^a^ • Stimulate somatostatin secretion^a^	(19, 20)
β_2_AR	Rhodopsin	Adrenaline; Noradrenaline	G_s_; G_i/o_; β-arrestin 1	• Stimulate glucagon secretion^a^ • Stimulate somatostatin secretion^a^	(19, 20)
β_3_AR	Rhodopsin	Adrenaline; Noradrenaline	G_s_; G_i/o_	• Enhance insulin secretion^b^	(21)
PAC_1_	Secretin	PACAP	G_s_; G_q/11_	• Enhance insulin secretion^b^ • Stimulate glucagon secretion^a^	(22, 23)
GLP1R	Secretin	GLP1	G_s_	• Inhibit cytokine-induced apoptosis of β-cells^c^ • Inhibit glucagon secretion^b^	(14)
GIPR	Secretin	GIP	G_s_	• Inhibit cytokine-induced apoptosis^c^ • Induce insulin production regulated by inflammation^b^	(14, 24)

*The GCPRs expressed in the pancreatic islet receive circulatory signals and exert their functions on hormone-secreting cells, initiating the autocrine and/or paracrine regulations between pancreatic cells. The primary transduction mechanisms are listed at the first row followed by secondary transduction mechanisms (if any)*.

a *Initiate both autocrine and paracrine regulations*.

b *Initiate paracrine regulations*.

c *Initiate autocrine regulations*.

Interestingly, apart from circulatory hormones, energy sources like free fatty acids (FFAs) can also act as signaling molecules. FFAs are obtained from dietary fat. Depending on the length of the carbon chain, FFAs bind to a multitude of GPCRs which are known as FFA receptors (FFARs), including G protein-coupled receptor 41 (GPR41) and GPR43 that bind to short chain FFAs, GPR84 for medium chain FFAs, GPR40 and GPR120 for long-chain FFAs, and GPR119 for long-chain FFAs and cannabinoid (26). These FFARs are differentially distributed in tissues and they signal through different G proteins for energy homeostasis. For example, GPR40 (also known as FFAR1) is expressed in human islets at levels comparable to those of GLP1R. At fasting glucose level, palmitate can enhance the secretion of glucagon and insulin via GPR40 on α- and β-cells (27), and this positive regulation is primarily mediated via the G_q/11_ signaling pathway (28). In contrast, GPR119 is expressed predominantly in β-cells. The binding of long-chain FFAs to GPR119 can increase the intracellular cAMP levels via G_s_ stimulation of adenylyl cyclase (AC) and promotes glucose-stimulated insulin secretion (29). Another example of organ crosstalk is between islet and the adipose tissues, which is mediated by adipose-derived signaling molecules (adipokines). Chemerin, one of the adipokines, was found to regulate glucose-stimulated insulin secretion and improve glucose tolerance via its receptor ChemR23 in mouse. The transduction mechanism of ChemR23 is coupled to the G_i/o_ pathway, while the functional relevance of the other two receptors of chemerin (GPR1 and CCRL2) remains unclear (17).

The pancreatic islet is additionally regulated by signals from the central nervous system (CNS). Multiple studies have demonstrated the roles of circadian clocks in key metabolic tissues, including liver, pancreas, white adipose, and skeletal muscle (30). In mammals, the suprachiasmatic nuclei (SCN) express a robust rhythm of electrophysiological activity that controls the secretion of melatonin by the pineal gland, with the diurnal variation in melatonin being crucial for synchronizing the circadian rhythm (31). The expression of type 1 and type 2 melatonin receptors (MT_1_R and MT_2_R) in the human islets has been confirmed by molecular and immunocytochemical approaches (32). Upon ligand binding, MT_1_R and MT_2_R suppress intracellular cAMP production via G_i/o_ proteins and reduce insulin secretion. The MT_2_R can also inhibit insulin secretion by suppressing the guanylate cyclase/cyclic guanosine monophosphate (GC/cGMP) pathway (33). A strong functional link between *MTNR1B* (encode MT_2_R) and type 2 diabetes risk was further established by Bonnefond et al (34), and an inhibitory effect of melatonin on somatostatin secretion has recently been demonstrated in a human pancreatic δ-cell line (35). Like melatonin, other classical neurotransmitters can also act as neurohormones to modulate pancreatic responses, and these include noradrenaline and adrenaline that inhibit pancreatic hormone secretion. Several subtypes of α_2_-adrenoceptors (α_2A_AR, α_2B_AR, and α_2C_AR) and β adrenoceptors (β_1_AR, β_2_AR, and β_3_AR) are known to be expressed in the pancreatic islet (36). The α_2A_AR on β-cells is important for G_i/o_-mediated inhibition of insulin secretion. Although less studied in humans, agonists of α_2A_AR can prevent excess insulin release (37) and variants of α_2A_AR are apparently associated with type 2 diabetes (38). Contrastingly, G_s_-coupled β-adrenoceptors have opposing effects, resulting in enhanced insulin secretion (39). The reduced β_2_AR expression may contribute to the age-related decline of glucose tolerance in mice (40). It has also been suggested that β_1_/β_2_ARs can increase somatostatin levels in mice via a G_s_-independent pathway composed of β-arrestin 1 and ERK1/2 (20). Overall, the available evidence supports the notion that G_s_-coupled receptors facilitate insulin and somatostatin secretion, whereas G_i_-coupled receptors tend to oppose these responses. However, co-activation of G_i_- and G_q_-coupled receptors was reported to have a synergistic stimulation on cytokine production (41). The mechanism of the observed synergism is presumably mediated via Gβγ-responsive isoforms of phospholipase Cβ (PLCβ2/3), which enable Gβγ dimers released from G_i_-coupled receptors to further stimulate PLCβ2/3 (42). Thus, synergistic action in regulating pancreatic hormones by GPCRs represents a distinct possibility which should be examined.

### GPCRs for Mediating Autocrine/Paracrine Regulations

Upon receiving various input signals, islet cells in turn secrete autocrine/paracrine molecules, a great number of which modulate the activity of neighboring cells through GPCRs (Table 2). Almost all endogenous GPCR ligands identified in the pancreas activate more than one type of receptor in the islet (9), suggesting that a ligand is able to trigger a variety of GPCRs present on multiple cell types, thereby diversifying the signaling event and inferring a robust paracrine regulatory mechanism. Among all the endocrine cell types within the islet, the paracrine interactions between α-, β-, and δ-cells have been proposed for a long time, with the somatostatin-secreting δ-cells providing essential negative feedback to both insulin and glucagon release, while the glucagon-secreting α-cells positively regulate insulin and somatostatin secretion (Figure 2) (53). Among the five human somatostatin receptor subtypes (SSTR_1−5_), only SSTR_1_, SSTR_2_, and SSTR_5_ show high expression levels in islet cells. SSTR_1_ and SSTR_2_ are selectively expressed on β-cells and α-cells, respectively. SSTR_5_ is highly expressed on both β- and δ-cells, and is moderately expressed on α-cells (54). Inhibition of glucagon and insulin secretion from the islet is primarily mediated by SSTR_2_ (43) and SSTR_5_ (44), respectively. Although all SSTRs are G_i/o_-coupled, SSTR_2_ and SSTR_5_ can additionally signal through G_q/11_ proteins. However, the G_q/11_ transduction mechanism in regulating pancreatic hormone secretion remains to be fully elucidated. One potential pathway may involve the activation of nuclear factor κB (NFκB) which regulates inflammation and cell survival. This is in agreement with the demonstrated ability of SSTR_2_ to activate NFκB via G_q_ family proteins (55), and that NFκB has been implicated in fatty acid-induce β-cell dysfunction (56). In contrast, α-cells work as a positive regulator. Acetylcholine released by α-cells stimulates insulin secretion by β-cells via the muscarinic M_3_ and M_5_ receptors (M_3_R and M_5_R), as well as somatostatin secretion by δ-cells through M_1_R (46). Interestingly, M_1_R, M_3_R and M_5_R are all coupled to the G_q/11_ pathway. Apart from the paracrine feedback system, α-cells have a glucagon autocrine feedback loop. Glucagon secreted by the α-cells can upregulate its own expression, the process of which is mediated by the glucagon receptor (GCGR) via G_s_-dependent signal transduction (49). Since the release of glucagon is stimulated by a lack of glucose, this kind of positive feedback may help to optimize the hormonal output response under less favorable energy conditions. In general, pancreatic hormone secretion is modulated by intracellular cAMP level. Stimulation of cAMP production by G_s_-coupled receptors leads to hormone secretion, while activation of G_i/o_-coupled receptors oppose this effect.

**Table 2:** Examples of GPCRs in the pancreatic islet for regulating pancreatic hormone secretion.

**GPCR**	**Class**	**Ligands**	**Transduction mechanism**	**Functions**	**References**
SSTR_2_	A	SomatostatinCortistatin	G_i/o_; G_q/11_	• Inhibit glucagon secretion^b^	(43)
SSTR_5_	A	SomatostatinCortistatin	G_i/o_; G_q/11_	• Inhibit glucagon secretion^b^ • Inhibit insulin secretion^b^	(43, 44)
M_1_R	A	Acetylcholine	G_q/11_	• Enhance insulin secretion^b^ • Stimulate somatostatin secretion^b^	(45, 46)
M_3_R	A	Acetylcholine	G_q/11_	• Enhance insulin secretion^b^ • Stimulate glucagon secretion^a^	(46, 47)
M_5_R	A	Acetylcholine	G_q/11_	• Enhance insulin secretion^b^	(46)
D2R	A	Dopamine	G_i/o_; β-arrestin 2	• Inhibit insulin secretion^a^	(48)
GCGR	B	Glucagon	G_s_; G_q/11_	• Up-regulate glucagon expression^c^ • Enhance β-cell function and mass^b^	(49, 50)
GABA_B_R	C	γ-Aminobutyric acid	G_i/o_	• Inhibit insulin secretion^a^	(51, 52)
mGluR3	C	L-glutamic acid	G_i/o_	• Enhance insulin secretion^b^	(51)
**mGluR5**	C	L-glutamic acid	G_q/11_; G_s_; G_i/o_	• Enhance insulin secretion^b^	(51)

*The GPCRs exert their functions on hormone-secreting cells via autocrine and/or paracrine regulations. The primary transduction mechanisms are listed at the first row followed by secondary transduction mechanisms (if any)*.

a *Mediate both autocrine and paracrine regulations*.

b *Mediate paracrine regulations*.

c *Mediate autocrine regulations*.

## GPCRs for Regulating Energy Homeostasis in the Brain

The brain modulates the endocrine system in response to external environment. The effect of circulatory hormones, in turn, can regulate brain chemistry and function. Similar to peripheral endocrine glands, stimulation of the brain by circulatory inputs can trigger a sophisticated autocrine/paracrine feedback network to generate an integrated output signal that regulates the body. However, the feedback network is more complex in the brain, and thus more difficult to determine the particular type of GPCR that stimulates or suppresses the release of hormones or neurotransmitters, as synaptic signal transduction and membrane potential need to be taken into account, the latter of which can be modulated not only by Gα subunits, but also by Gβγ dimers. Examples of GPCRs for regulating energy homeostasis in the brain are listed in Table 3.

**Table 3:** Examples of GPCRs for regulating neuoendocrine functions in brain.

**GPCR**	**Class**	**Ligands**	**Transduction mechanism**	**Functions**	**References**
GHSR_1a_	Rhodopsin	Ghrelin	G_q/11_; G_i/o_; G_12/13_	• Modulate reward circuits^b^ • Stimulates appetitive processes^a^	(57, 58)
CCK_2_R	Rhodopsin	CCK	G_q/11_	• Thermoregulation^b^ • Increase body weight and water consumption^b^	(59)
Y_1_R	Rhodopsin	NPY; PP; PYY	G_i/o_	• Mediate hyperphagic effects^b^	(60)
Y_2_R	Rhodopsin	NPY; PP; PYY	G_i/o;_ G_q/11_	• Inhibit NPY production^a^ • Stimulate POMC production^a^	(61, 62)
Y_4_R	Rhodopsin	NPY; PP; PYY	G_i/o;_ G_q/11_	• Inhibit NPY production^a^ • Stimulate POMC production^a^	(63, 64)
Y_5_R	Rhodopsin	NPY; PP; PYY	G_i/o_	• Mediate hyperphagic effects^b^	(60)
MC_3_R	Rhodopsin	ATCH; MSHs; ASP; AgRP	G_s_	• Inhibit adiposity^b^	(65)
MC_4_R	Rhodopsin	ATCH; MSHs; ASP; AgRP	G_s_	• Mediate hyperphagic effects^b^ • Inhibit adiposity^b^	(64, 65)
CRFR1	Secretin	CRH; urocortin 1	G_s;_ G_q/11_	• Stimulate ACTH secretion^a^ • Mediate stress responses^b^	(66)
MCH_1_R	Rhodopsin	MCH	G_s_; G_i/o_; G_q/11_	• Meidate orexigenic effects^b^	(67)

*The GPCRs influence energy homeostasis by sensing circulatory inputs and mediating autocrine/paracrine interactions between brain cells. The primary transduction mechanisms are listed at the first row followed by secondary transduction mechanisms (if any)*.

a *Mediate both autocrine and paracrine regulations*.

b *Mediate paracrine regulations*.

### From Circulatory Inputs to Neuroendocrine Signals

In the case of energy homeostasis, it is important for the brain to sense the level of metabolic substances in order to regulate energy usage. In the CNS, the hypothalamus is considered as an essential area where the nervous system and the endocrine system meet. Metabolic signals such as glucose, insulin, cholecystokinin (CCK), pancreatic polypeptides (PP), and peptide YY (PYY), and ghrelin have all been found to modulate the activities of the hypothalamic arcuate nucleus (ARC), hence altering food intake and metabolism (68). Among these signals, ghrelin has received intense interest as it can upregulate food intake while the majority acts in the opposite manner (68). Circulatory ghrelin is mainly produced by the gastric X/A-like cells of oxyntic stomach mucosa under hunger situation (69). Ghrelin receptor type 1a (GHSR_1a_) is highly expressed in the ARC and ventromendial nucleus (VMN) of the hypothalamus (70). By activating phospholipase C (PLC) via G_q/11_ protein, GHSR_1a_ triggers the release of neuropeptide Y (NPY) that exerts paracrine effects (which will be discussed later). The heteromerization of GHSR_1a_ with other GPCRs further broadens its downstream responses. Various studies have demonstrated that GHSR_1a_ specifically forms dimers with the SSTR_5_ (71), dopamine D_1_ and D_2_ receptors (D_1_R and D_2_R) (72, 73), melanocortin-3 receptor (MC_3_R), and 5-hydroxytryptamine receptor 2C (5HT_2c_R) (74). The protomers within these dimers exhibit different signaling effects that range from facilitation, inhibition, and even modification of the pathways (75). Thus, heteromerization of GHSR_1a_ represents a putative mechanism to regulate food intake and energy balance. Ghrelin treatment is also found to alter the dopamine and acetylcholine receptor gene expression in the mesolimbic reward circuitry (76). In contrast to ghrelin, many circulatory signals tend to down-regulate the energy intake. PYYs are stimulated during meal intake by the presence of nutrients (especially fat) in the small intestine. The Y_2_ receptor (Y_2_R), which is coupled to G_i/o_ and G_q/11_ proteins, is critical in mediating the effects of PYY_3−36_ on reducing adiposity and feeding (77). The expression of Y_2_R can be found throughout the CNS, within the nodose ganglion and on vagal afferents, thus the feeding effects of PYY_3−36_ is possibly mediated through central, vagal activation, or a combination of both (77). The pattern of c-fos neuronal activation following peripheral administration of PYY_3−36_ further suggests the involvement of Y_2_R in the ARC (77).

### GPCR-Mediated Autocrine and Paracrine Regulations in the Hypothalamus for Energy Homeostasis

**Figure 3:**
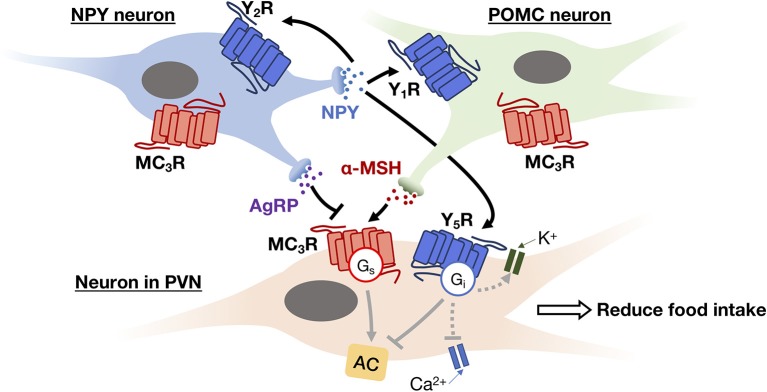
An example of autocrine and paracrine interactions between NPY neuron (blue) and POMC neuron (green) in the ARC and their downstream effector neuron (i.e., neuron in PVN; beige). NPY secreted by NPY neuron can trigger G_i_ signaling pathway to inhibit Ca^2+^ channel activity and activate GIRK current via its receptors (Y_1_R, Y_2_R, and Y_5_R) in both autocrine and paracrine manners, thereby inhibiting the release of neurotransmitters. While AgRP and α-MSH secreted by NPY neuron and POMC neuron, respectively, act as antagonist and agonist of MC_3_R. The activation of MC_3_R can reduce food intake via the feeding circuit in the hypothalamus.

Within the hypothalamus, ghrelin can be synthesized by different hypothalamic nuclei including dorsomedial, ventromedial, paraventricular nucleus (PVN), and the ARC. In the brain, ghrelin mainly acts on the presynaptic terminals of NPY neurons and stimulates the activity of arcuate NPY as demonstrated by electrophysiological recordings (78). NPY neuron is known to inhibit the release of pro-opiomelanocortin (POMC) from POMC neurons using neurotransmitter GABA, at the same time it can stimulate the secretion of melanin-concentrating hormone (MCH) and hypocretin/orexin from the lateral hypothalamus via NPY. The inhibition of POMC together with the secretion of MCH and hypocretin/orexin appears to increase food intake and reduce metabolic rate by acting on the PVN of hypothalamus. This network of paracrine regulation adjust metabolism through multiple output pathways that eventually enhance appetite (68). All NPY receptors in the human hypothalamus, including Y_1_R, Y_2_R, Y_4_R and Y_5_R, activate the G_i/o_-signaling pathway. Depression of Ca^2+^ channel and increasing G protein coupled inwardly rectifying potassium channel (GIRK) currents are also observed upon G protein activation by NPY (79). The Y_1_R expressed in the ARC has been suggested to mediate the hyperphagic effect of NPY (60), while the ARC NPY expression is negatively regulated in an autocrine manner via presynaptic Y_2_R and Y_4_R present in NPY neurons (62, 63). The perifornical part of the lateral hypothalamus, which is considered as the feeding center, contains a high density of Y_5_R that mediates NPY-induced hyperphagia (60). Besides, NPY neurons produce another orexigenic peptide, the agouti-related peptide (AgRP), as an endogenous antagonist to the MC_3_R and MC_4_R (80). MC_3_R and MC_4_R are G_s_-coupled receptors present in various hypothalamic nuclei which mediate the neuronal circuits to reduce food intake and increase energy expenditure (81, 82), and their endogenous agonist, α-melanocyte stimulating hormone (α-MSH), is synthesized by POMC neurons in the ARC using precursor POMC protein (81). An example of autocrine and paracrine interaction between the NPY neuron, the POMC neuron, and their downstream effector neuron is shown in Figure 3. Additionally, the POMC neurons can produce another anorexic peptide known as CART or cocaine and amphetamine related transcript (81). Although the receptor for CART remains to be characterized, the activation of G_i/o_ signaling pathway has been observed upon CART application (83). The paracrine communication between NPY/AgRP and POMC/CART neurons can further modulate downstream neuronal activity via GABAergic signaling (84). Overall, with the participation of GPCRs, the autocrine/paracrine communication between the orexigenic NPY/AgRP neuron and the anorexigenic POMC/CART neuron regulates a variety of physiological and behavioral events to maintain energy balance.

## GPCRs for Bridging the Autocrine/Paracrine Network and for Signal Integration

While an autocrine signal is an amplifier or a brake for message transmission, a paracrine ligand is a tool to disperse the message from one cell type to the others. It has been observed that many ligands can be recognized by multiple GPCR subtypes that are expressed in different cell types. Hence, once a paracrine signal is generated in an organ, various cell types could be differentially activated or inhibited by the same signal via different subtypes of GPCRs. Using pancreatic islet as an example again, somatostatin produced by δ-cells can simultaneously target subtypes of SSTRs on α-cells, β-cells and δ-cells, thereby inhibiting the secretion of both glucagon, insulin and somatostatin itself (53) (Figure 2). The biological setting of SSTR distribution indeed bridges the autocrine/paracrine feedback network. Since multiple cellular responses can be elicited by the same ligand, it results in a synchronized signal propagation. Apart from the pleiotropic regulation by a ligand, some GPCRs recognize more than one type of ligands so that enabling the integration of diverse signals. For example, MC_3_R in ARC can bind MSHs as well as AgRP with the former acting as agonists and the latter as an antagonist. Competition between these two ligands for the MC_3_R population on the cell surface will ultimately determine the downstream cellular response (Figure 3). Biased GPCR signaling, wherein an agonist preferentially triggers a specific downstream pathway, may offer additional avenues to modulate autocrine/paracrine signaling. GPR40 is a pharmacological target to increase insulin secretion in type 2 diabetes, and a synthetic ligand TAK-872 has been shown to induce a β-arrestin-biased pathway instead of G protein signals that are typically elicited by native ligands such as palmitate and oleate (85). It is generally accepted that biased agonists have great clinical potentials, but their importance in the regulation of autocrine/paracrine signaling remains to be fully appreciated. The ultimate hormonal output from an endocrine cell is influenced by multiple pathways that are mediated by different receptors. Pancreatic β-cells express G_s_-coupled GLP1R and G_i/o_-coupled SSTR_5_, and when both GLP1 and somatostatin are simultaneously presented to the β-cells, GLP1R would give activation signal while the SSTR_5_ sends inhibition signal to adenylyl cyclase (14, 44). The net effect of which represents an integrated outcome that depends on the relative strengths of the two signals. Furthermore, a single cell can express over a 100 types of GPCRs. The overlapping downstream pathways allow further integration of different messages. Since most cells express numerous GPCRs that receive ligands including circulatory hormones and other molecules including autocrine and paracrine factors, the final outputs from the cell are hence adjusted by signaling molecules in multiple contexts, producing more than one type of signal output, with specific cellular responses regulated by groups of receptors. Therefore, the cell-specific expression profile of GPCRs contributes to the incredibly complicated interactions: for cells to implement the autocrine/paracrine feedback networks, and to integrate various signals for fine-tuning the output. GPCRs such as GLP1R, GHSR, and MC_4_R are popular pharmaceutical targets for diseases related to energy homeostasis, especially for obesity and diabetes (86). With recent advancements in structural determination and computational techniques, it is envisioned that major strides in our understanding of ligand/receptor interactions may lead to the identification of novel compounds that act as orthosteric, allosteric, or biased ligands of GPCRs. However, it is tremendously important to fully elucidate the pharmacological capabilities of individual GPCRs in the context of signaling networks, as adverse drug effects are often associated with indirect modulations of physiological systems. Hence, a thorough understanding of the diversity and complexity of GPCR signaling is critical for successful therapeutic development of GPCR ligands.

## GPCRs Mutations and Perturbation of Energy Homeostasis

Since GPCRs are intricately involved in the regulation of energy homeostasis in both the CNS and peripheral organs, it is not surprising that mutations of GPCRs are found to be associated with endocrine diseases (3). For example, the MT_2_R mutations in ligand binding and G protein activation are associated with type 2 diabetes (87), and MC_4_R deficiency is prevalently found in obesity (88) (please refer to sections GPCRs for Sensing Circulatory Inputs and GPCR-Mediated Autocrine and Paracrine Regulations in the Hypothalamus for Energy Homeostasis, respectively). The pathogenesis of which may be attributed to the dysregulation of GPCRs in intercellular communication, can impair cell-to-cell interactions and the autocrine/paracrine feedback loops that are critical for maintaining homeostasis. As illustrated in animal models, mice with GCGR null mutation (GCGR^−/−^) display supraphysiological glucagon levels, increased proglucagon expression and increased pancreatic and circulating GLP1 (89). The GCGR^−/−^ mice also exhibit reduced adiposity and leptin levels while having normal body weight, food intake, and energy expenditure. These disease phenotypes are associated with postnatal enlargement of the pancreas and hyperplasia of islets, which is mainly due to α-cell, and to a lesser extent, δ-cell proliferation (89). Likewise, the ablation of GCGR delays β-cell differentiation and perturbs the proportion of β- to α-cells in embryonic islets, inhibits the progression of α-cells to maturity in adult mice, as well as affecting the expression of several β-cell-specific genes (90). It has been noted that an augmentation in both islet number and in the rate of proliferation of α- or β-cells led to increased cell mass of the islet (including the δ-cell mass) in those mutant mice (90). These findings in mice suggest that glucagon participates in an autocrine/paracrine feedback loop that regulates the proportion of the different endocrine cell types in islets, the number of islets per pancreas, and development of the mature α-cell phenotype. It was subsequently demonstrated that the overexpression of GCGR increases β-cell mass (89). In humans, a study reported that a homozygous P86S mutation of the human GCGR is associated with hyperglucagonemia, α-cell hyperplasia, and islet cell tumor α-cell hyperplasia (91). This receptor mutant displayed lower affinity to glucagon and decreased cAMP production of the cells at physiological glucagon level. It is likely that the insufficient glucagon signaling perturbs the negative feedback on α-cell proliferation by other islet cells. In this case, the disease is not simply due the alteration of their signaling functions, the influence of malfunction is stepped up by the disruption of autocrine/paracrine feedback loop, which eventually affect the endocrine organ and the greater biological system. Indeed, the engagement of various autocrine/paracrine regulations in human physiology and pathophysiology has been well-recognized over the years. The therapeutic implications of autocrine/paracrine modulators that act on GPCRs are being explored in not only endocrine diseases such as obesity and diabetes (92), but also in cancers (93) and heart failure (94). A number of studies suggested that drugs that can propagate autocrine/paracrine signals could further enhance the efficacy of the therapy. Nevertheless, although GPCRs are one of the most popular pharmacological targets, very little is known with regard to the pathophysiological mechanisms of GPCRs that regulate autocrine/paracrine communications. By better understanding the roles of GPCRs in autocrine/paracrine regulations, one might reveal new avenues for therapeutic interventions against various diseases.

## Concluding Remarks

It is known that GPCRs participate in almost every process in the regulation of energy homeostasis as well as other physiological processes that are not mentioned in this review. In the process of maintaining homeostasis, an intricate network of autocrine/paracrine feedbacks is often involved. Due to the pleiotropic property of many GPCR ligands, the diversity of GPCRs and their subtypes, and the potential involvement of multiple intracellular signaling pathways and crosstalks, it is rather difficult to distinguish the physiological roles of autocrine and paracrine factors in a tissue. Tools like co-culture transwell system (95) and the more recently established microcavity platform (96) have been used to study the interactions of selected cell types, which help to unmask autocrine/paracrine communications between cells. Moreover, advances in single cell sequencing techniques enable the identification of GPCR expression profile on different cell types, thus providing a clear mapping of possible GPCR autocrine/paracrine pathways, and it may even be possible to discern between the two mechanisms. By profiling the expression of GPCRs in various cell types within a tissue or organ, we may begin to elucidate the complexity of autocrine/paracrine regulatory pathways under physiological as well as pathophysiological conditions. Subsequent knockdown or knockout experiments can then be conducted to confirm the importance of potential GPCR autocrine/paracrine pathways in healthy and disease states. Given that GPCRs represent highly amenable targets for drug development, deciphering their roles in autocrine/paracrine regulations of human physiology may provide new insights for treatment regimens against complex diseases such as diabetes.

## Author Contributions

All authors listed have made a substantial, direct and intellectual contribution to the work, and approved it for publication.

### Conflict of Interest Statement

The authors declare that the research was conducted in the absence of any commercial or financial relationships that could be construed as a potential conflict of interest.
